# Clinical features, radiological findings, and prognostic factors for primary intracranial chordoid meningioma

**DOI:** 10.3389/fneur.2022.1002088

**Published:** 2022-11-10

**Authors:** Danyang Jie, Zhiyong Liu, Wenbo He, Shumin Wang, Haibo Teng, Jianguo Xu

**Affiliations:** ^1^Department of Neurosurgery, West China Hospital of Sichuan University, Chengdu, China; ^2^West China School of Medicine, Sichuan University, Chengdu, China

**Keywords:** meningioma, chordoid meningioma, clinical features, surgical resection, prognosis

## Abstract

**Objectives:**

Chordoid meningioma (CM) is an infrequent histologic subtype of meningiomas. Owing to its low occurrence, this subtype has been rarely described. Our subject was to explore the clinical features, radiological characteristics, and prognostic factors of primary intracranial chordoid meningioma.

**Methods:**

We reviewed the medical records and collected follow-up information of 34 cases who had been surgically treated and histologically diagnosed with CM at the Department of Neurosurgery, West-China Hospital of Sichuan University, from January 2009 to December 2021.

**Results:**

Among all 7,950 meningioma cases, the proportion of primary intracranial CM was 0.43% (34/7,950). The median diagnosis age was 47 (ranging from 12 to 74) and the gender ratio (male to female) was 2.1:1. For radiological features, heterogeneous enhancement, skull base, and ventricular localization, cystic degeneration and dural tail sign were common in CM cases. In treatment, gross total resection (GTR) was achieved in 22/34 cases (64.7%) and subtotal resection (STR) was achieved in 12/34 cases (35.3%). Further, 11/34 patients (32.4%) had received postoperative adjuvant radiotherapy (RT). The follow-up duration ranged from 4 to 157 months after operation. The progression rate was 20.7% (6/29) and the median of PFS was 38 months. By survival analysis, accepting adjuvant radiotherapy and achieving GTR were correlated with longer progression-free survival for prognosis.

**Conclusion:**

CM is a rare subtype of meningiomas. In our series, it mainly involved adults and did not show a predilection for women compared with meningiomas in general. For a better prognosis, gross total resection and postoperative adjuvant radiotherapy are recommended. Nevertheless, due to the restriction of the series sample, patients lost for follow-up and inherent biases of a retrospective study, more cases and a shorter follow-up duration are needed for better management of chordoid meningioma.

## Introduction

Meningiomas have made the most common primary tumor in the central nervous system (CNS) (1). According to the 2021 WHO Classification of Tumors of the Central Nervous System, meningiomas were still divided into three grades with 15 different subtypes or variants, and grade II and III meningiomas are classified for their high tendency to relapse (2–4).

Chordoid meningioma, a rare subtype of grade II, was initially defined as a subtype of meningiomas for its chordoma-like pathologic features by Kepes et al. (5, 6). The incidence of CM was reported to be 0.32–1.0% of all intracranial meningiomas with an incidence of 4.4 per 1,00,000 individuals per year (7–11). Due to the low incidence, the clinical, radiological, pathological, and prognostic features of CM still remain unclear and controversial. This study reviewed the clinical manifestations, radiological features, and surgical information for 34 cases pathologically diagnosed with primary intracranial CM in West China Hospital of Sichuan University (About 2,800 CNS tumors operations per year), and explored prognostic factors for this rare subtype.

## Methods

### Patient population

According to our exclusion and inclusion criteria, we reviewed the medical records of West China Hospital of Sichuan University from 2009 to 2021 and identified 34 cases diagnosed with primary intracranial chordoid meningioma. The pathological diagnosis of CM was based on the WHO 2021 Classification (5th version). The inclusion criteria were: precise histologically diagnosis of CM, intracranial location of lesions, and complete medical record information; the exclusion criteria were recurrent CM, extracranial CM, pathological diagnosis with other varieties of meningioma subtype or tumor species, and patients undergoing adjuvant radiotherapy before surgery. Histopathological evaluation was performed to validate the diagnosis of CM. The acquisition of medical records and patient information was approved by the West China Hospital Ethics Committee.

### Clinical and radiological data acquisition

The clinical manifestations were acquired from the hospital information system (HIS). Duration from hospitalization to discharge was defined as hospital day. The image data was collected from Picture Archiving and Communication System (PACS). The quality of life was assessed by Karnofsky Performance Scale (KPS) score. The pre-KPS and post-KPS were appraised by two authors, respectively at admission and discharge. Two experienced radiologists assessed the radiological features on MRI or computed tomography (CT) scans separately. Tumor size was defined as the maximal diameter on an MRI scan. The shape of the tumor was categorized to be spherical or irregular. Globular and ellipsoidal tumors were identified as spherical shape and lobulated, branched, dumbbell-like tumors were classified as irregular ones. Peritumoral edema was assessed on T2-weight and Flair scan sequences of MRI. According to the tumor basal attachment, the location of the tumor was divided into three major groups: the skull base group mainly included the anterior skull base, sellar region, sphenoid ridge, petroclival, temporal fossa, cerebellopontine angle (CPA), foramen magnum, and clivus; the convexity group mainly included cerebral or cerebellar hemispheres lesions, falx, and sagittal sinus CM were also included; and intraventricular group incorporated tumors occurring in the lateral, third, or fourth ventricle. Homogeneous enhancement was defined as uniformly enhanced in >90% of tumors on enhanced MRI scans.

### Surgical information and pathological data

All patients involved in our study had undergone neurosurgery by routine craniotomy approach in our center. The extent of resection (EOR) was evaluated by the Simpson resection grade standard and we defined levels I and II as GTR and levels III and IV as STR. The short-termed and long-termed postoperative complications were acquired via medical records and follow-up data.

The diagnosis of CM was performed pathologically based on WHO 2021 Classification and retrospectively reviewed by two experienced pathologists, both of whom have performed neuropathological diagnosis independently for 10 years. The immunohistochemical indicators introduced into the statistical analysis were determined according to the results of univariate analysis and previous studies.

### Outcome

The follow-up information was acquired by telephone or outpatient approaches after discharge. The loss rate of follow-up was 14.7% (5/34). History of postoperative adjuvant radiotherapy (RT), postoperative complications, post-KPS, and progression-free survival (PFS) were recorded at the first time of follow-up (always 3 months after discharge). PFS was defined as the duration from surgery to tumor progression. Tumor regression included tumor regrowth and tumor relapse. The tumor regrowth was defined as residual tumor getting growth after STR and tumor relapse was defined as tumor recurring after GTR. Hospital day (HOD) refers to the duration from accepting surgery to discharge.

### Statistical methods

All statistical analysis was performed by SPSS (version 24.0, IBM Corporation, Armonk, NY, USA). We introduced the following factors to prognostic analysis: age of surgery, gender, radiological features, history of adjuvant radiotherapy, EOR, pre- and post-KPS value, and pathological features. Kaplan-Meier curve was utilized to figure PFS and a log-rank test was conducted to assess whether there was a statistical difference between curves. Cox proportional hazards model was conducted for univariate analysis. A *p*-value < 0.05 was considered as statistical significance. For continuous variables, we reported the mean and standard deviation of normal distribution. The median together with (25% quartile, 75% quartile) were used to report non-normal distribution. Proportions were utilized for categorical variables.

## Results

### Incidence and demographic features of CM

From January 2009 to December 2021, there were 7,950 patients with meningioma undergoing operation in the Department of Neurosurgery, West China Hospital of Sichuan University. There were only 38 cases diagnosed with intracranial chordoid meningioma pathologically. After excluding four recurrent patients, there were 34 (0.43%) cases involved in our study. For these 34 patients, the median diagnosis age was 47 (33, 60) years (ranging from 12 to 74) and the sex ratio of male to female was 2.1:1 (23/11).

### Clinical characteristics

The median interval from initial symptoms to admission and undergoing surgical intervention was 3 months (1, 18). The most frequent symptoms were headache or dizziness (20 cases, 58.8%), followed by visual or auditory impairment (14 cases, 41.2%), limb weakness (10 cases, 29.4%), and epilepsy (3 cases, 8.8%). The tumor was incidentally discovered without any symptoms during physical examination in 2 cases (5.9%).

### Radiological findings

In our series, preoperational enhanced MRI or CT scan images could be acquired in 30 cases and the other 4 cases did not receive preoperational radiological examination in our institution for being examined in other hospitals, without available radiological records for tumor features. Among the 30 cases with a radiological examination, 10 cases were located in convexity, 20 cases in the skull base, and 4 cases in a ventricle (3 cases in the lateral ventricle and 1 case in the fourth ventricle). The median of the maximal diameter of CM was 36.7 mm (26.1, 51.9), with a wide interval ranging from 19 to 82 mm. Seventeen cases were characterized as having an irregular shape (56.7%) and 15 cases were identified as unclear tumor-brain boundary (50%). Peritumoral edema were found in 10 cases (33.3%) with the extent of edema ranging from 12.7 to 48.4 mm. Cystic degeneration was found in 5 cases (16.7%). As a significant radiological feature of meningioma on enhanced MRI scan, a dural tail sign was identified in 20 cases (76.9%). CM with homogenous enhancement on the T1-weighted scan comprised 46.2% (12/26) and heterogenous enhancement comprised 53.8% (14/26). All the demographic, clinical, and radiological features are listed in Table 1.

**Table 1:** Demographic, clinical, radiological charecteristics of CM.

	**Overall (*N* = 34)**
**Demographic features**	
**Sex**	
Male	23 (67.6)
Female	11 (32.4)
Age (median, (P25, P75))/year	47 (33, 60)
**Clinical features**	
Headache or dizziness	20 (58.8)
Visual or auditory impairment	14 (41.2)
Limb. weakness	10 (29.4)
Epilepsy	3 (8.8)
Asymptomatic	2 (5.9)
**Radiological features**	
**Location**	
Non-skull base	14 (41.2)
Skull base	20 (58.8)
Diameter (median, (P25, P75))/mm	36.7 (26.1, 51.9)
**Shape**	
Spherical	13 (43.3)
Irregular	17 (56.7)
**Boundary**	
Clear	15 (50.0)
Unclear	15 (50.0)
Peritumoral brain edema	10 (33.3)
Dural tail sign	20 (79.9)
**Enhancement**	
Homogeneous	12 (46.2)
Heterogeneous	14 (53.8)
Cystic degeneration	5 (16.7)
Calcification	7 (23.3)

### Treatment and outcome

In our series, all 34 patients were treated surgically and the neurosurgical strategy was chosen due to the localization of tumor basal attachment. GTR was completed in 22 cases (64.7%) and STR in 12 cases (35.3%). Eleven patients (32.4%) received postoperative adjuvant RT. In our series, there was 1 postoperative mortality (2.9%) caused by acute brain hernia secondary to postoperative cranial hemorrhage and other 10 postoperative morbidities (29.4%): two pneumonia cases (5.9%), two intracranial infection cases (5.9%), two post-operational epilepsy cases (5.9%), one patient experienced hydrocephalus (2.9%), receiving trepanation and drainage of the ventricle, there were two cranial nerve impairment cases (5.9%) and one cranial hemorrhage (2.9%) receiving emergency craniotomy. After intravenous use of antibiotics, anti-epileptic drugs, or emergency surgery, all these patients were alleviated on discharge. No significant difference between EOR or tumor location and the presence of postoperative complications (Fisher's precision probability test, *p* = 0.714, *p* = 0.467) was found. The pre-KPS score ranged from 50 to 100 with a median of 60, and the post-KPS score ranged from 30 to 100 with a median of 80. There were 20 cases (69.0%) that had an improvement on the KPS after surgery while 9 cases (31.0%) saw a reduction. The median of HOD was 7.5 days (5, 13), ranging from 3 to 46 days.

### Histopathological features

Pathology on H&E staining showed that CM cells were composed of spindle or epithelioid cells characterized by myxoid basophilic matrix, arranged in chordoma-like clusters. Lymphoplasmacellular infiltrate was also a histopathological feature of CM. Immunohistochemically, the median of the MIB-1 index was 5%, and the detailed treatment and histopathological characteristics are listed in Tables 2, 3.

**Table 2:** Treatment and outcomes of CM.

	**Overall (*N* = 34)**
**Treatment and outcomes**	
**Extent of resection**	
GTR	22 (64.7%)
STR	12 (35.3%)
Accepted radiotherapy	11 (32.4%)
**Pre-KPS**	
<80	29 (85.3%)
≥80	5 (14.7%)
**Post-KPS**	80 (55,100)
<80	12 (41.4%)
≥80	17 (58.6%)

**Table 3:** Pathological features of CM.

	**Overall (*N* = 34)**
**Immunohistochemical features**	
**MIB-LI**	
<5	15 (50%)
≥5	15 (50%)
**S-100**	
Negative	26 (89.7%)
Positive	3 (10.3%)
**PR**	
Negative	3 (13.0%)
Positive	20 (87.0%)
**CD34**	
Negative	17 (100%)
Positive	0

^*^PR, progesterone receptor.

### Prognosis

After excluding five patients (14.7%) lost to follow-up, we analyzed the follow-up data of the rest 29 cases. The median follow-up duration was 44 months (16, 102), ranging from 4 to 157 months. Six patients experienced tumor progression, including one relapse patient and five regrowth patients, displaying a progression rate of 20.7%, with a median of 38 months (13, 93). The 2-year PFS rate was 78% and the 5-year was 71%. In progressive cases, two patients accepted re-operation while the other four patients declined surgery or radiotherapy. After re-operation, one patient experienced multiple recurrences and chose conservative treatment then. Among these six progressive or recurrent patients, four patients (66.7%) died of tumor progression.

**Figure 1:**
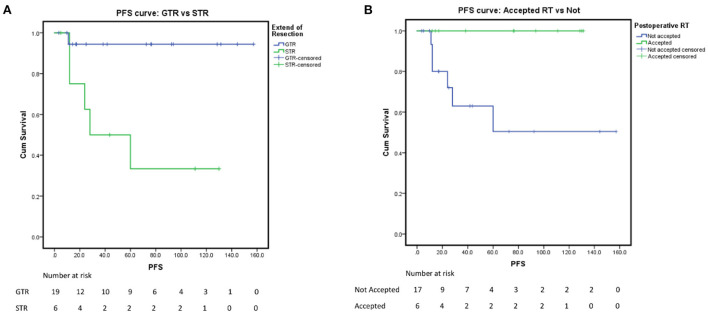
Progression-free survival rate. **(A)** PFS of the extent of resection: GTR vs. STR, *p* = 0.005. **(B)** PFS of postoperative radiological therapy: accepted vs. not, *p* = 0.021.

**Figure 2:**
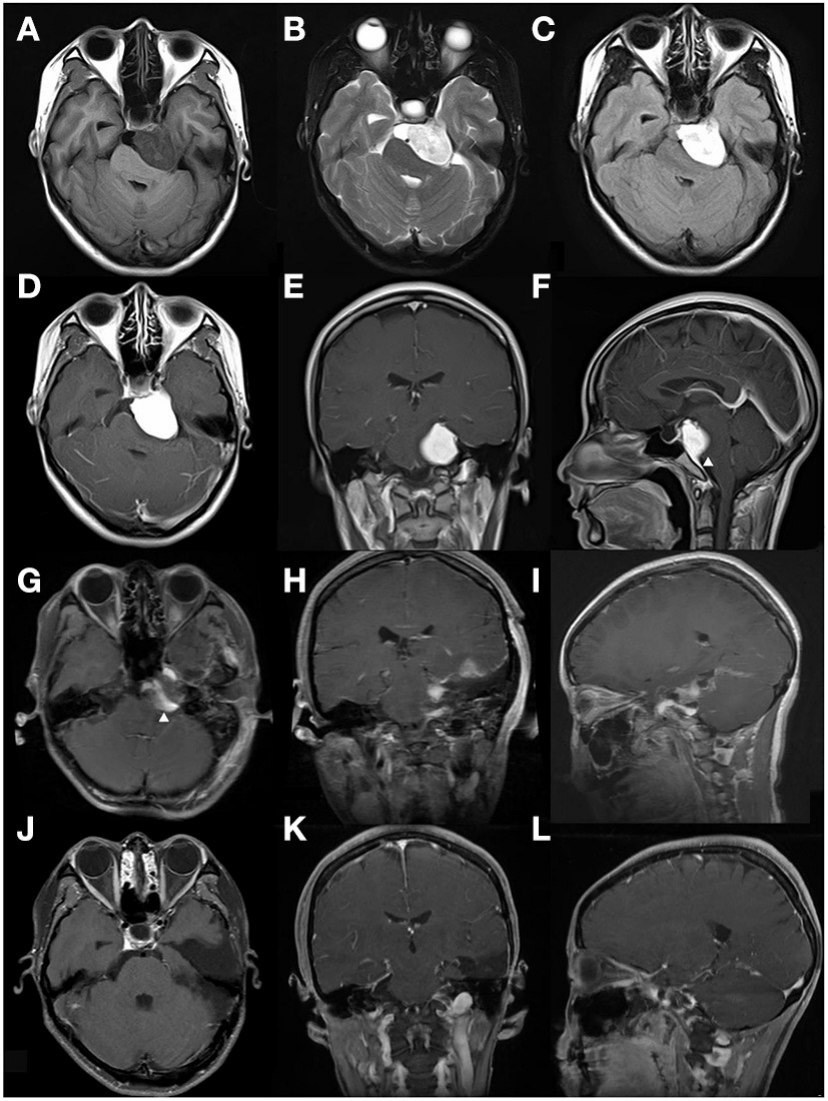
This case is a 26-year-old female patient (case 5 in Table 4). The tumor was found incidentally during a physical examination without any symptoms or signs. **(A–C)** Preoperative MRI scan showed a left CPA region tumor, defined as relatively heterogeneous signal intensity on T1, T2, and Flair-weighted MRI scan. The tumor shape was identified to be regular. **(D–F)** Enhanced MRI scan showed the lesion was a homogeneous enhancement. A dural tail sign was obviously evident arrowhead in **(F)**. **(G–I)** On the 2nd day after the operation, an enhanced-MRI scan was performed and identified an incomplete resection arrowhead in **(G)**. 10 days after surgery, this patient accepted radiotherapy (Gamma knife). **(J–L)** Enhanced MRI scan performed 8 years after surgery indicated that the residual tumor disappeared and no regression occurred.

**Figure 3:**
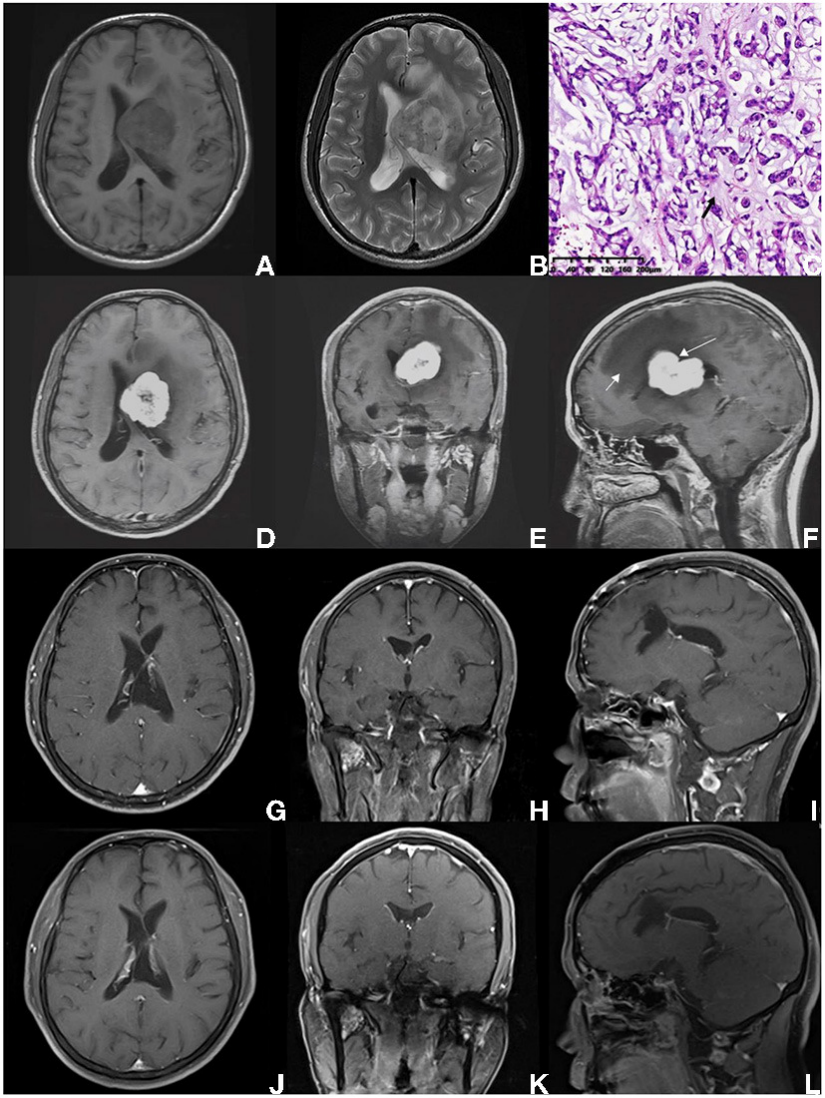
A 38-year-old male pathologically diagnosed with CM, who presented with persistent headache for 4 months without other symptoms or signs (case 1 in Table 4). **(A,B)** The intraventricular mass was heterogeneous, with hypointense on T1-weighted imaging, and presented hyperintense on T2-weighted MRI scan, surrounding with brain edema. **(C)** On HE staining, CM was composed of epithelioid cells characterized by myxoid and partly vacuolated matrix. The black arrow indicates the mucoid matrix. **(D–F)** Preoperative enhanced MRI scan showed an intraventricular lesion with heterogeneous enhancement. The shape of the tumor was identified to be sightly tabulated long arrow indicates in **(F)**. Peritumoral brain edema was also found (short arrow indicates). **(G–I)** MRI scan performed on the 2nd day after surgery suggested the GTR was achieved. According to patient willingness, postoperative radiotherapy was not performed. **(A–L)** After 11-year-follow-up, an enhanced MRI scan showed no progression.

In our analysis for the prognostic factors of CM, we introduced patient sex, age of onset, tumor location, tumor size, tumor shape, peritumoral edema, tumor shape, tumor boundary, calcification, cystic degeneration, pre-KPS, HOD, the extent of resection, postoperative RT history, and Ki-67 into the univariate analysis. Kaplan-Meier survival analysis with log-rank test was performed for the possible prognosis according to univariate analysis. Achieving GTR and accepting adjuvant radiotherapy were related to a longer PFS (log-rank test, *p* = 0.005, *p* = 0.021, respectively Figures 1A,B) in the log-rank test. However, the other factors we optioned showed no significant influence on the PFS of our CM series. The multivariate analysis was not performed due to the low events number. A detailed list of patient data and factors analyzed in our study are presented in Tables 4, 5 and Figure 1. Besides, we presented two representative cases in Figures 2, 3.

**Table 4:** Data presentation of 34 cases with CM.

**No**	**Sex**	**Age**	**Radiological data**		**EOR**	**Histopathology**				**Radiotherapy**	**Progression**
			**Location**	**MRI or CT scan**		**Ki-67**	**PR**	**EMA**	**GFAP**		
1	M	38	Lateral ventricle	YES	GTR	2%	-	+	-	NO	NO
2	M	28	Convexity	YES	GTR	/	-	-	-	YES	NO
3	F	48	Foramen magnum	NO	GTR	5%	/	+	-	NO	NO
4	M	35	Anterior skull base	YES	GTR	/	/	+	-	NO	NO
5	F	26	Cerebellopontine angle	YES	STR	4%	/	+	-	YES	NO
6	M	44	Convexity	YES	GTR	4%	+	+	-	YES	NO
7	M	26	Convexity	YES	STR	8%	/	+	-	YES	NO
8	F	49	Convexity	YES	STR	10%	+	+	-	NO	NO
9	M	20	Fourth ventricle	YES	GTR	10%	/	+	-	YES	NO
10	F	47	Clivus	YES	GTR	/	+	+	-	NO	NO
11	M	71	Convexity	YES	GTR	3%	+	+	-	NO	NO
12	F	56	Anterior skull base	YES	GTR	/	/	/	/	YES	NO
13	M	43	Anterior skull base	YES	GTR	3%	+	+	-	NO	NO
14	M	68	Cerebellopontine angle	YES	GTR	8%	+	+	/	YES	NO
15	M	60	Lateral ventricle	NO	GTR	5%	/	+	-	YES	NO
16	M	34	Sellar region	YES	GTR	4%	+	+	+	NO	NO
17	M	57	Convexity	YES	GTR	10%	+	+	-	YES	NO
18	F	57	Anterior skull base	YES	GTR	5%	+	+	-	NO	NO
19	M	71	Anterior skull base	YES	GTR	3%	+	+	-	NO	NO
20	M	47	Clivus	YES	GTR	3%	+	+	/	NO	NO
21	F	33	Convexity	YES	GTR	5%	+	+	-	YES	NO
22	M	47	Anterior skull base	YES	GTR	3%	+	+	-	YES	NO
23	F	40	Clivus	YES	STR	5%	-	+	-	NO	NO
24	M	74	Sellar region	YES	STR	5%	+	+	-	NO	YES
25	M	47	Sphenoid ridge	YES	STR	2%	+	+	-	NO	YES
26	M	21	Cerebellopontine angle	NO	GTR	8%	/	+	+	NO	YES
27	M	72	Convexity	YES	STR	2%	+	+	-	NO	YES
28	M	12	Lateral ventricle	YES	STR	12%	/	+	-	NO	YES
29	F	21	Lateral ventricle	YES	STR	5%	/	+	-	NO	YES
30	F	33	Convexity	YES	GTR	8%	/	+	-	NO	LOST
31	M	45	Foramen magnum	YES	GTR	4%	+	+	-	NO	LOST
32	F	61	Anterior skull base	NO	STR	3%	+	+	-	NO	LOST
33	M	65	Temporal fossa	YES	STR	3%	+	+	-	NO	LOST
34	M	60	Sphenoid ridge	YES	STR	4%	+	+	-	NO	LOST

**Table 5:** Factors associated with PFS in CM patients.

**Variables**	**Univariate**		**Log-rank test**
	***p*-value**	**HR (95% CI)**	***p*-value**
Age (years) ≥55	0.940	/	/
Female	0.537	/	/
Skull-base location	0.946	/	/
Tumor size	0.887	/	/
Irregular tumor shape	0.384	/	/
Unclear boundary	0.674	/	/
Peritumoral edema	0.212	/	/
Calcification	0.901	/	/
Cystic degeneration	0.810	/	/
Pre-KPS ≥80	0.532	/	/
Hospital day	0.479	/	/
Accept radiotherapy	0.227	/	0.021
Gross total resection	0.028	11.163 (1.300-95.885)	0.005
Ki-67 ≥5%	0.533	/	/

## Discussions

### Incidence and clinical features

Meningiomas cause the most common primary CNS tumors (1, 12–14). Though the majority of meningiomas show a benign clinical manifestation, some rare variants are distinguished by more aggressive behavior and propensity for relapse. Initially described by Kepes et al. (6) CM has been classified to be a subtype of meningiomas. Because of its greater likelihood of recurrence and more aggressive behavior compared with other histology of meningiomas, CM has long been classified as WHO grade II. In previous studies, meningioma constituted ~39% of all CNS tumors (1) and CM only accounts for 0.32–1.0% of all meningiomas (6, 9–11, 15, 16). In our institution, primary intracranial CM accounts for 0.43% (34/7,950) of all meningiomas, paralleling the previous reports.

Meningioma has been known to have a female predominance (17, 18). The sex ratio (M:F) of CM ranged from 0.7 to 1.2 in previous literature (5, 15, 19). The ratio of 2.1 (23/11) in our series showed a predilection for men. Initially, when defined as an individual subtype of meningiomas by Kepes et al. CM was believed to have a preponderance of child cases and often be associated with systemic diseases (6, 20). Castleman syndrome (CS), a series of syndromes characterized by microcytic anemia, localized or disseminated lymphadenopathy, polyclonal hypergammaglobulinemia, and developmental retardation, was noted to be associated with CM in the pediatric population. Such systematic abnormalities were ascribed to an overproduction of interleukin 6 (IL-6), which could disappear after the removal of the tumor (6, 20–24). In our series, neither 33 adult cases nor 1 pediatric case were connected to systematic manifestations of CS or other hematologic disorders. The absence of CS or other systematic disorders in our series may be correlated with the low incidence of CM in children. Consistent with previous literature, we found that CM mainly occurred in adults rather than children (5, 15, 16, 19, 25–28). In this study, CM was mainly exhibited as mass effect such as headache (20/34), when the anatomical localizations were adjacent to cranial nerves or cerebrum function area, CM would present cranial nerves disorders (14/34), limb weakness (10/34), or epilepsy (3/34).

### Radiological features

In this study, the most common location of CM was the base of the skull (20/34, 58.8%), followed by convexity (10/34, 29.4%), and ventricle (4/34, 11.8%). In a previous systematic review involving 221 CM cases, localization of convexity was much more common than skull base (19). Additionally, compared with meningiomas in general, the intraventricular cases were more common in CM (11.8 vs. 0.5–3%) (29, 30). Such differencse may be caused by the selection bias of the positioning of our hospital as a central neurosurgery institution where more cases of the skull base or intraventricular underwent surgery. Our series presented a wide interval of tumor size (ranging from 19 to 82 mm), consistent with previous research (19, 31). Due to the limitation of the space of the skull base, the shapes of our series were most identified to be irregular (56.7%) and unclear tumor-brain interfaces (50%). Peritumoral edema was not mentioned much in preceding studies. The incidence of peritumoral edema in meningiomas ranged from 38 to 67.2% (32). For CM the proportion was from 26.7 to 77.8% in previous studies (16, 31, 33) and 33.3% in our series. In our series, CM cases with heterogeneous enhancement on T1-weighted imaging comprised 53.9% (14/26), which may be related to ischemia and necrosis, calcification, or cystic degeneration of the tumor. Additionally, cystic degeneration was found to be more common in CM than overall meningiomas (16.7% vs. 2–4%) (34–36), which may be caused by hemorrhage, necrosis, or glial response (37). The radiological features of chordoid meningioma in our study can be summarized as follows: localization predilection for skull-base and ventricle, high occurrence of cystic degeneration, irregular shape, unclear interface, heterogeneous enhancement, and dural tail sign.

### Treatment strategies and outcome

CM together with atypical meningioma and clear cell meningioma were classified into the grade II category of meningiomas for their relatively high recurrence rate (38). Previous large series have reported that achieving GTR might indicate a better prognosis (5, 6, 15, 31). Therefore, surgical resection, especially GTR, is the primary treatment and gold standard treatment for CM. While various factors hindered achieving GTR such as the complex anatomic location of the tumor, invasion of surrounding vital neurovascular structures, or immense volume of the tumor. When achieving GTR accompanies a high risk of serious complications, incomplete removal should be considered. In our series, GTR was performed in 22 cases (64.7%) with STR in 12 cases (35.3%) and that proportion was 78.2 and 21.8% in a systematic review involving 221 CM cases of Choy et al. in 2016 (19). Such difference may be caused by the adjacent complex structures with the predilection for skull base location in our series.

Due to the high recurrence of CM, 11 patients (32.6%) received postoperative adjuvant RT after surgical resection (nine GTR cases and two STR cases). In our institution, postoperative radiological therapy was recommended to WHO grade II and III meningioma patients systematically. All patients accepted RT within 1 month after discharge. While for the low adherence of patients coming from all over the country and other reasons, the rate of receiving postoperative RT was relatively low in this retrospective study. In previous studies, the benefit of postoperative adjuvant RT remains controversial (15, 16, 39, 40) thus more studies are needed to discuss the benefit of RT to patients.

### Pathological features

**Figure 4:**
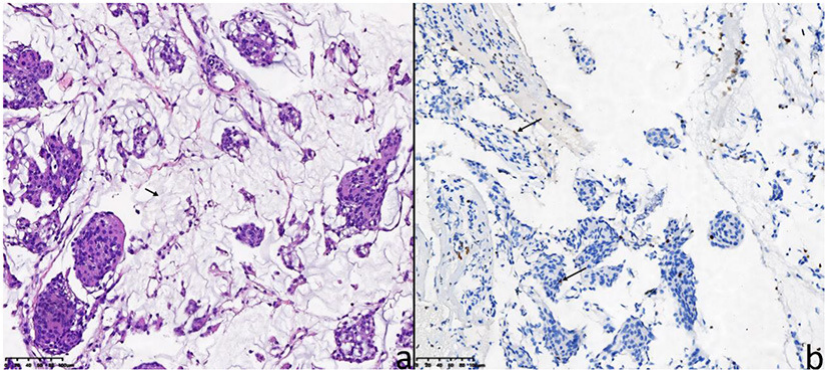
The pathological features of the 26-year-old female patient (case 5). **(a)** Hematoxylin-eosin staining showed chordoma-like epithelioid cells arranged in clusters with mucoid matrix (short arrow indicates mucoid matrix). **(b)** For the 26-year-old female patient, the Ki-67 proliferation index (MIB-1 antibody) was immunohistochemically 4% (long arrows).

As a rare subtype of meningiomas, CM was characterized by chordoma-like vacuolated cells and pale basophilic matrix (5, 41). These pathological features are presented in Figure 4 and the corresponding description. Due to the histological features of CM, the mucoid quality of the matrix may facilitate tumor cells to disseminate and result in a high recurrence rate after STR (15, 39, 42, 43).

Immunohistochemically, as a proliferation marker predicting the proliferative activity of meningioma, MIB-1 expresses in nearly all stages of cell cycle except G_0_ (44, 45). Whether the MIB-labeling index (LI) was correlated with the prognosis of CM was still controversial. Due to recent reviews, a high MIB-LI is correlated with a high risk of tumor recurrence (7). This study suggested that higher MIB-LI implicated the recurrence of the tumor and the increasing WHO grade of meningioma. Besides, male sex was identified as an independent risk factor for high MIB-LI (46, 47). In our study, MIB-LI was available in 30 cases, with 15 cases <5% positive and 15 cases ≥5% positive. The difference between the two levels of MIB-LI was not of statistical significance (*p* = 0.538), and such risk factor of sex was not found. For the wide range of MIB-LI from various institutions, the value for predicting the prognosis of CM cases was uncertain (5, 16, 33). A previous study involving 30 CM cases showed a MIB-LI range of 1–10%, without relation to prognosis (15). Therefore, more cases, stricter standards, and better statistical approaches are needed. Compared with commonly positive in Grade I meningiomas and used as a risk predictive factor, progesterone receptor (PR) are used to be a diagnostic marker for higher grade meningioma (48, 49). Glial fibrillary acidic protein (GFAP) was found positive in 2 of 31 cases (6.5%) and to be important for the differential diagnosis of CM from gliomas and chordomas (41). GFAP was regarded to be only expressed in gliosarcomas and hemangioblastomas (50), but some studies have reported that GFAP may be sporadically positive in a few CM patients. Due to previous studies, invaded brain tissue surrounded by tumor may facilitate the positivity of GFAP. Compared with strong expression in gliomas and chordomas, the positivity of GFAP in CM is mainly dura-based and marginal (10, 31, 39, 51, 52). S-100 protein is helpful to distinguish meningioma from schwannoma (53). As a kind of single-pass transmembrane sialomucin protein, the positivity of CD34 is considered to be correlated with high microvessel density which is a risk factor for the prognosis of CM (54–56). In our series, all 17 cases available for CD34 were negative. These markers are mainly employed for the diagnosis of meningioma at present and the combination of multiple markers was proved to be more sensitive and specific for distinguishing meningioma from their morphological mimics (50).

### Prognosis

In our series, we reviewed the follow-up data of 29 (85.3%) cases after excluding 5 patients (14.7%) lost. The overall progression rate was 20.69% (6/29), in accordance with previous studies (16–42%) (5, 6, 15, 16). In a larger series, Yang et al. (31) reported a recurrence rate of 31.5% (17/54), and Wang et al. (15) reported a 19% progression rate (5/27). The most concerning prognostic factors included EOR (log-rank test, *p* = 0.005) and adjuvant post-operational radiotherapy (log-rank test, *p* = 0.021). Yang et al. (31) reported that only irregular tumor shape together with STR implicated the worse prognosis in univariate analysis (*p* = 0.014, *p* = 0.004), without the significance of adjuvant RT (*p* = 0.154).

EOR has been widely confirmed to be a critical prognostic factor of CM (5, 15, 19, 31). In our series, progression occurred, respectively in 5% of cases with GTR and 55.6% of cases with STR. According to the low progression rate and our result, GTR should be encouraged to be achieved on the premise that vital structures must be protected.

Under what circumstances adjuvant RT is required remains controversial (15, 16, 39, 40). In our institution, receiving adjuvant radiotherapy after STR is considered to be much safer and more beneficial. Additionally, post-operational RT is also recommended for all grade II and III meningiomas in our institution. There is no doubt that larger prospective studies are essential to evaluate the prognostic value of adjuvant RT for CM.

In neurosurgical operations, tumor localization may exert an influence on the difficulty and duration of the surgery, and the extent of resection also could be impacted. Similarly, age, tumor shape, tumor boundary, and tumor size may also have an influence on EOR and prognosis. Therefore, multivariate analysis is always conducted to control confounding bias among diverse factors. Nevertheless, The progression event of 6 cases was insufficient for multivariate analysis, which is essential for clarifying the prognostic factors for CM, this is a limitation of our study. Although without statistical significance in univariate analysis, postoperative RT was still considered as a prognostic factor for CM due to its *p*-value in log-rank test and clinical experience in our institution. In a previous study (31), STR was uniformly the only risk factor for recurrence (*p* = 0.008, HR 4.191). Limited by the relatively small sample size in our study, more cases are definitely needed to be involved in multivariate regression analysis and systematic review for more specific risk factors and better management of CM.

### Limitations

Our research had several limitations. Firstly, this study was conducted retrospectively so that the inherent biases and their impact on the study exist. Secondly, the sample of case series requires to be expanded and the follow-up duration needs to be prolonged to obtain more specific risky or protective factors for better management of CM. Thirdly, some radiological or pathological data were unable to acquire due to the lack of systematic management for CM cases. Furthermore, the number of patients accepting radiotherapy was small thus survival analysis may be biased, so in the future, we need to make more standard postoperative RT criteria for CM patients. Finally, patients of our institution came from all over the nation, which resulted in relatively poor compliance for postoperative follow-up.

## Conclusion

CM is a rare subtype of meningiomas. In our series, it mainly involved adults and did not show a predilection for women compared with meningiomas in general. There were some radiological features of CM in our series: localization predilection for skull-base and ventricle, high occurrence of heterogeneous enhancement, cystic degeneration, and dural tail sign. For a better prognosis, gross total resection and postoperative adjuvant radiotherapy are recommended. Nevertheless, due to the restriction of the series sample, patient loss to follow-up, and inherent biases of retrospective study, more cases, and lower follow-up duration are needed for better management of chordoid meningioma.

## Data availability statement

The raw data supporting the conclusions of this article will be made available by the authors, without undue reservation.

## Ethics statement

Ethical review and approval was not required for the study on human participants in accordance with the local legislation and institutional requirements. Written informed consent from the patients/participants or patients/participants' legal guardian/next of kin was not required to participate in this study in accordance with the national legislation and the institutional requirements.

## Author contributions

Conception and design: JX, DJ, WH, and ZL. Data acquisition and analysis: DJ and SW. Writing and review: DJ. Research supervision: ZL and JX. Reviewed submitted version of manuscript and approved the final version of the manuscript on behalf of all authors: JX. All authors contributed to the article and approved the submitted version.

## Funding

This work was supported by the General Program of the National Natural Science Foundation of China (Grant No. 82173175), the Knowledge Innovation Program of the Chinese Academy of Sciences (Grant No. JH2022007), and 1·3·5 projects for disciplines of excellence–Clinical Research Incubation Project, West China Hospital, Sichuan University (Grant No. 2020HXFH036).

## Conflict of interest

The authors declare that the research was conducted in the absence of any commercial or financial relationships that could be construed as a potential conflict of interest.

## Publisher's note

All claims expressed in this article are solely those of the authors and do not necessarily represent those of their affiliated organizations, or those of the publisher, the editors and the reviewers. Any product that may be evaluated in this article, or claim that may be made by its manufacturer, is not guaranteed or endorsed by the publisher.
